# Creative arts and digitial interventions as potential tools in prevention and recovery from the mental health consequences of adverse childhood experiences

**DOI:** 10.1038/s41467-022-35466-0

**Published:** 2022-12-22

**Authors:** Kamaldeep Bhui, Sania Shakoor, Anna Mankee-Williams, Michaela Otis

**Affiliations:** 1 Department of Psychiatry, Nuffield Department of Primary Care Health Sciences and Wadham College at the University of Oxford. Oxford Health and East London NHS Foundation Trusts. World Psychiatric Association Collaborating Centre, Oxford, UK; 2 Centre for Psychiatry and Mental Health, Wolfson Institute of Population Health, Queen Mary University of London, London, UK; 3 Falmouth University, Falmouth, UK; 4 Imperial College; North West London Applied Research Collaborative, London, UK

**Keywords:** Public health, Psychology

## Abstract

Adverse childhood experiences (ACEs) can harm mental health across the lifespan and reduce life expectancy. We provide a commentary of evidence on the health impacts, and how creative arts and digital interventions may support prevention and recovery.

Adverse childhood experiences refer to verbal, sexual, or physical abuse; emotional or physical neglect; parental separation or incarceration, problem drug and alcohol use in the family, domestic violence or mental illness in the family; additional experiences that are harmful include bullying, poverty, peer rejection, racism, death and multiple traumatic losses, community violence, food shortages, harsh experiences in care, poor academic performance, and living in unsafe environments, prior to age 18 years^1,2^. Community data from worldwide surveys of adults report high prevalence rates of adverse childhood experiences (75% of the population), with a mean of three such experiences^3^. Other prevalence studies break this down by type of adverse experience: emotional (29.1%), physical (22.9%), and sexual (9.6%) abuse; as well as physical (16.3%) and emotional neglect (18.4%)^4^. By the age of eight, 7 in 10 children report at least one ACE and 1 in 10 children report three or more adverse childhood experiences^1,2^.

ACEs have mental health impacts across the lifespan^1^ by disrupting neurodevelopment, leading to social, emotional and cognitive impairment, and increasing risky behaviours, disability and social exclusion^5^. The underlying mechanisms include loss of trust, poor relational competence due to misjudging threats and rewards, disrupted autobiographical memories as a vulnerability for future mental health problems, changes in the regulation of fight and flight responses regulated by the amygdala leading to aggression or passivity and cycles of victimisation^6–11^. The importance of adverse childhood experiences are recognised world-wide, albeit surveillance systems and interventions will need local adaptation and implementation^12,13^.

Evidence suggests that cumulative exposures, rather than individual experiences, have the most significant negative outcomes including shortened life expectancy by up to 20 years^1,2^. Trauma exposed adolescents are more likely to develop complex mental illnesses including major depression, conduct disorder, alcohol dependence, self-harm, suicide attempts, and post-traumatic stress disorders (PTSD)^5^. There are also additional difficulties in education, work, and life-course transitions ^5^. Consequently adverse childhood experiences have a detrimental social, health and economic cost^14^. However, not all young people who experiencing adversity develop these poor outcomes in later life. System-wide or individual resiliency characteristics can alleviate the long-term effects of adversity^15^. In order to develop interventions, we need to understand these mechanisms (Fig. 1), and how individual and wider environmental factors impact mental health and resilience^1^.

**Fig. 1:**
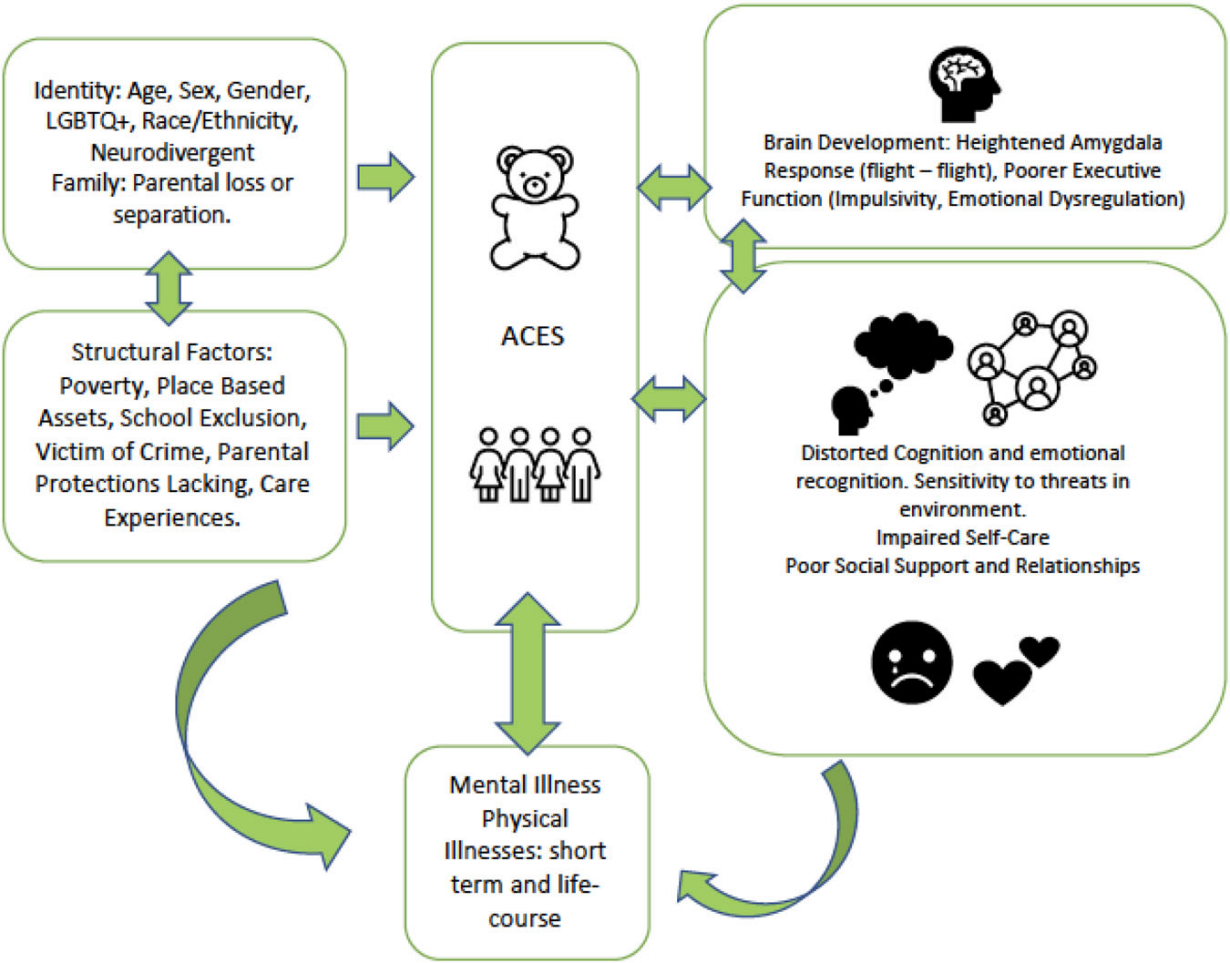
Pathways adversity to mental illness. Adverse Childhood experiences (ACES) affect young people from many demographic and social contexts (represented by the teddy bear) and involve adults/communities in terms of protections and therapeutic responses (i.e., structural factors). These two interact and the individual mechanisms affect families and communities.

This commentary aims to discuss (1) What are the risks of mental illness and symptoms amongst young people who have experienced adversity? (2) How do intersectional influences impact on the magnitude of these risks? (3) How effective are arts-based interventions in supporting young people exposed to adversity? (4) How effective are digital intervention in supporting young people exposed to adversity?

## The risk of mental health illness and symptoms amongst young people who have experienced adversity?

Adverse childhood experiences increase vulnerability for poor physical, mental and socio-educational outcomes throughout the lifespan^5,14^. Meta-analytic evidence suggests a dose-response effect, whereby exposure to four or more adverse experiences contributes to greater odds of poor health outcomes^16^. Data were pooled from 37 studies, indicating adverse experiences led to a 2–3 fold increase in the odds of smoking, heavy alcohol use, poor self-rated health, cancer, heart disease, and respiratory disease. There was also a 4–6 fold higher odds of risky sexual behaviour and a 7–8 fold greater odds of exposure to interpersonal violence as victim or perpetrator^16^. Associations have also been reported for mental health conditions, including major depressive disorder^14^; anxiety disorders^14^ and psychosis^17^.

Efforts to explore the aetiological relationships between adverse childhood experiences and the wellbeing of survivors have focused on the timing and type of adversities. Emotional abuse appears to increase the incidence of depressive disorders more than physical abuse; by contrast, physical abuse is associated with increased incidence of problematic drug use more so than emotional abuse^18^. Furthermore, the timing of adversities differentially impacts on the development of psychopathology. Exposure earlier in life (ages 0–5) predicted more symptoms of anxiety and depression in adulthood, than exposure at older ages (ages 6–8); the latter better predicted adult behavioural problems (e.g. substance misuse)^19^.

One explanation for the sensitivity of age of exposure to adversity and consequential impact on mental health comes from the neuroscience literature. Studies have shown that exposure to adversities is associated with a significant reduction in grey-matter volume in the primary visual cortex amongst those who were survivors of childhood sexual abuse^20^, and in the left anterior and posterior cingulate cortex and bilateral precuneus (regions involved in self- evaluation and self-awareness) amongst those who were exposed to emotional abuse^21^. These reductions contribute to the altered development of sensory systems which process and interpret stress in response to adversity. This may contribute to distorted perceptions, cognitions and emotional regulation abilities that are associated with many mental illnesses.

A key mechanism for poor outcomes, is that adversities are implicated in the development of emotion dysregulation and biases in emotion recognition^5^. In comparison to peers who have not experienced adversities, individuals who have a history of adversities report lower levels of emotional understanding^22^ and distorted perceptions and greater sensitivity to negative facial expressions associated with anger and fear^23^. This can impact on relationships and levels of social support and communications with teachers and employers, leading to more challenges in progressing in life. These emotional impacts in turn may contribute to the development of mental health disorders rooted in emotional disturbances (i.e. anxiety, depression and PTSD)^24^. Similarly adverse childhood experiences are related to cognitive deficits such as the maladaptive formation of cognitive attributional styles^25^, hypersensitivity to threat in ambiguous situations and distortions in social information processing^5^. For example, being repeatedly exposed to adversities may contribute to the development of maladaptive cognitive processing, whereby individuals internalise the belief that the consequences of adversities are fixed and stable, have only negative consequences, and are attributable to their own conduct and behaviours. These cognitive schema contribute to the development of psychopathology such as depression^26^.

Thus, adversities in childhood can lead to multiple mental health conditions with including post-traumatic stress, depression, psychoses, and dissociative symptoms, and may be complicated by poor self-care, self-medication (alcohol and substance misuse), and efforts to seek help or avoid help given this would become a reminder of the traumatic events. The mechanisms (Fig. 1) include direct and indirect pathways, and inflammatory responses are implicated in partially mediating the impacts of adversity^5,27^.

## Intersectional risks of mental illness amongst young people who have experienced adversities in childhood

Although adversities affect people across ethnicity, race, neurodiversity and socioeconomic groups, the intersecting effects of such factors are cumulative drivers of increased vulnerabilities. The frequency, types and experiences of adversities are differentially distributed across these groups and associated with disproportionate burdens of trauma amongst some communities^28^. The risk of adversities is greater amongst those who come from low income or single-parent households, or reside in unsafe neighbourhoods and lack access to health care^15^. In one study, data from the USA suggests that Black and Latino youth were exposed to a greater risk of experiencing ACEs in comparisons to their white peers^29^. As children and families belonging to racial and ethnic minority groups are more likely to live in low-income neighbourhoods and experience greater socioeconomic difficulties^30^, the interconnected systems-wide effect of disadvantage may increase vulnerabilities to the effect of adverse childhood experiences on mental and physical health and dampen recovery^30^. Albeit limited, there is some evidence to suggest similar patterns of elevated risks amongst racial and ethnic minorities in the United Kingdom^31^. Incidence rates of childhood maltreatment are one and a half times greater amongst those from a Black minority community in comparison to those from a white community^31^. Incidence rates are five-fold greater amongst those from the most socioeconomically deprived backgrounds in comparison to those from the least deprived in the UK^31^. In addition, neurodiverse individuals with Autism Spectrum Disorder (ASD) and ADHD are more likely to have experienced multiple adverse childhood experiences (ACEs) than neurotypical peers^32^.

Intersectional factors contribute to the high prevalence of adverse childhood experiences globally^4^. These intersectional factors should be considered in the design of interventions to reduce barriers in service use and therapeutic impacts. More experiential research is needed to move away from the traditional approach of exploring adversity from a universal perspective and move towards developing a more nuanced understanding of experiences in specific populations, carrying specific intersectional vulnerabilities.

## The effectiveness of arts-based intervention for young people exposed to adverse experiences in childhood

Creative arts such as music, dance, drama and visual arts, and arts-based therapies are emerging as important approaches in the treatment of childhood trauma. Arts based research and interventions permit gradual exploration of adverse experiences, where the creator exercises control over disclosure, and non-verbal disclosure or awareness grows, leading to narration at deeper levels so offering an ethical and safer process that is less likely to lead to distress or trigger traumatic symptoms^33–35^. Evidence suggests that arts-based interventions improve sense of achievement, self-confidence, self-esteem, social skills, conflict resolution, problem solving, relationships and sense of belonging amongst adolescents^35^. Equally, these creative activities have been successful in improving mental health. Studies have found amongst young people who have experienced sexual abuse, music therapy^36^ and group art therapy^37^ have been effective in reducing depression, anxiety and PTSD symptoms.

It is hypothesised that the participatory and creativity elements of arts-based interventions can contribute to resilience and recovery in a number of ways. For example, art has been found to elicit narrative and facilitate exposure to traumatic cues in a non-threatening and non-invasive manner, this in turn allows for the vocalisation of affective states and reduction in depressive and anxious symptoms^37^. Arts-based interventions have also been shown to modulate emotional responses to environmental cues and affect mood, through influencing emotional expression and regulation. Using creative practices to express and deal with negative emotions has been found to foster self-esteem, positive relationships and skills for overcoming adversity^34^. In addition, the group settings that creative arts-based intervention are often engaged in have the additional benefit of facilitating trust and disclosure amongst the young people partaking. This can provide an opportunity for individuals to realise that they are not alone in their experiences, have a sense of belonging and find peer-support.

## The effectiveness of digital intervention in supporting young people exposed to adversities in childhood

Young people are often described as ‘digital natives’. This aptitude coupled with the ubiquitous nature of digital technologies (i.e. smart phones and wearables) in modern life, support the therapeutic shift to digital health interventions (DHIs) such as apps, and virtual reality environments. These can increase the accessibility and support available to vulnerable and hard to reach individuals. These advantages alongside anonymity, instant feedback and cost-effectiveness^38^, suggest that digital interventions have the potential to be more helpful and overcome barriers to service use. Furthermore, stigma, fears about confidentiality, shame, financial costs^39^ all undermine help seeking; digital approaches may also address the limited numbers of skilled clinicians and therapists in rural areas or in countries with less health spend (low and middle income countries). Young people seek emotional and social support and more flexible help, which digital technology can offer^40^. Indeed, there is evidence that young people exposed to ACEs use digital media more often than other young people^41^.

Digital interventions can be used to screen and monitor mental health symptoms, and overcome physical barriers that affect attendance and retention at care services^42^. Young people prefer Digital Health Interventions (DHIs) which include features such as videos, limited text, personalisation and the ability to connect with others^43^. Thus the shift to using DHIs to support young people exposed to trauma has the potential to be revolutionise care experiences.

Digital intervention in the form of serious games may have a positive impact for young people internalising symptoms of mental health^44^. Augmented and virtual reality adapted games have been used and found to be helpful for health care design and delivery, in physical and mental health conditions^44^. Similarly, digital story telling has been implemented as a narrative intervention when working with survivors of trauma^45^. By integrating personalised digital images, text, audio narration, and music, digital story telling supports the processing, organising, and integrating of traumatic memories. New narratives are created where the trauma is disentangled from the associated negative thoughts, reminders and emotions. However, there is a dearth of research in this area and more is needed to determine effectiveness and optimisation and personalisation for implementation. Indeed, adding creative arts methods as content, or trying to replicate creative processes in digital design may add value. However, this again needs evaluation.

Although these approaches show promise for providing therapeutic support to young people exposed to adversities^44^, there are some concerns. Firstly, the experiences of virtual realities are dependent on gathering patient experiences to ensure this ‘real-world knowledge’ is included in the design to better support the ability to use and feel connected with virtual environments. The accuracy of the representation of real-world factors in the virtual environment can affect the ability to transfer learnt skills back into real world situations. Secondly, through digitalising interventions for traumatic experiences, therapeutic mechanisms such as active listening, empathic understanding and exploration of personal stories may be lost. There may still be a need for supervision, guided use of such interventions, and support to transition from immersive therapeutic technologies, back into ordinary life. Thirdly, there are ethical concerns about triggering disturbing experiences without appropriate support by a clinician or therapist, thus highlighting the need to always take participant safety into consideration when working with vulnerable individuals. Lastly, it can be challenging to preserve sufficient personalisation options in the up scaling of interventions delivered via digital platforms. Public and patient involvement at the time of development and testing of digital designs for interventions, could help overcome some of these concerns. For example, including elements of supervised individual and community support and incorporating creative elements in the generating digital content, could offer some reassurance and safeguards. For some, personal therapeutic support may be the only mechanism by which recovery is possible, thus identifying for whom digital interventions may work and how is an important research priority.

## Conclusions

There is a growth in digital platforms for mental health, and increased attention to designing virtual realities for therapeutic purposes. In the UK this is ever more pertinent due to the increased pressures NHS service provisions experience when providing care to young people who have experienced trauma, and especially so in the current climate of COVID-19 and limited capacity in health systems^46^. Currently there is some evidence on the effectiveness of digital interventions for young people’s mental illness, but much more is needed. Further research will need to evaluate the implementation, effectiveness and cost-effectiveness of interventions for a range of mental disorders (e.g. ADHD, psychosis and eating disorders), and comorbidities which are more common following multiple adversities. The interventions will need to address and be responsive to a number of social contexts. We anticipate future research will produce and evaluative a taxonomy for digital mental health interventions for adverse childhood experiences. It is important to consider the mechanistic drivers of trauma related mental illness, and recovery processes. Arts-based interventions show promise for recovery from trauma due to the creative and interactive elements, and such processes may be designed into digital interventions. Indeed, digital interventions already contain much arts content, so the distinction may not be as clear cut. A number of ethical dilemmas need exploration alongside the intervention design and delivery. The approach we propose is to engage young people in the co-design and implementation of research on digital health interventions.

## References

[1] NHS Highlands. *Adverse Childhood Experiences, Resilience, and Trauma Informed Care* (The Annual Report of the DIrector of Public Health, 2018).

[2] Bethell CD (2017). Methods to assess adverse childhood experiences of children and families: toward approaches to promote child well-being in policy and practice. Acad. Pediatr..

[3] Pace CS (2022). The Adverse Childhood Experiences-International Questionnaire (ACE-IQ) in community samples around the world: a systematic review (part I). Child Abus. Negl..

[4] Sethi, D. et al. *European Report on Preventing Child Maltreatment: World Health Organization* (Regional Office for Europe, 2013).

[5] Jaffee SR (2017). Child maltreatment and risk for psychopathology in childhood and adulthood. Annu. Rev. Clin. Psychol..

[6] Neil L (2022). Trust and childhood maltreatment: evidence of bias in appraisal of unfamiliar faces. J. Child Psychol. Psychiatry.

[7] Armbruster-Genc DJN (2022). Altered reward and effort processing in children with maltreatment experience: a potential indicator of mental health vulnerability. Neuropsychopharmacology.

[8] Goemans, A., Viding, E. & McCrory, E. Child maltreatment, peer victimization, and mental health: neurocognitive perspectives on the cycle of victimization. *Trauma Violence Abuse*10.1177/15248380211036393 (2021). 10.1177/15248380211036393 PMC10009486 34355601

[9] Gerin MI (2019). Heightened amygdala reactivity and increased stress generation predict internalizing symptoms in adults following childhood maltreatment. J. Child Psychol. Psychiatry.

[10] McCrory EJ, Gerin MI, Viding E (2017). Annual Research Review: Childhood maltreatment, latent vulnerability and the shift to preventative psychiatry—the contribution of functional brain imaging. J. Child Psychol. Psychiatry.

[11] McCrory E, Viding E (2010). The neurobiology of maltreatment and adolescent violence. Lancet.

[12] Alhowaymel F, Kalmakis K, Jacelon C (2021). Developing the concept of adverse childhood experiences: a global perspective. J. Pediatr. Nurs..

[13] Anda RF (2010). Building a framework for global surveillance of the public health implications of adverse childhood experiences. Am. J. Prev. Med.

[14] Bellis MA (2019). Life course health consequences and associated annual costs of adverse childhood experiences across Europe and North America: a systematic review and meta-analysis. Lancet Public Health.

[15] Berger LM (2004). Income, family structure, and child maltreatment risk. Child. Youth Serv. Rev..

[16] Hughes K (2017). The effect of multiple adverse childhood experiences on health: a systematic review and meta-analysis. Lancet Public Health.

[17] Varese F (2012). Childhood adversities increase the risk of psychosis: a meta-analysis of patient-control, prospective-and cross-sectional cohort studies. Schizophrenia Bull..

[18] Norman RE (2012). The long-term health consequences of child physical abuse, emotional abuse, and neglect: a systematic review and meta-analysis. PLoS Med..

[19] Kaplow JB, Widom CS (2007). Age of onset of child maltreatment predicts long-term mental health outcomes. J. Abnorm. Psychol..

[20] Tomoda A (2009). Childhood sexual abuse is associated with reduced gray matter volume in visual cortex of young women. Biol. Psychiatry.

[21] Heim CM (2013). Decreased cortical representation of genital somatosensory field after childhood sexual abuse. Am. J. Psychiatry.

[22] Shipman K (2000). Emotion management skills in sexually maltreated and nonmaltreated girls: a developmental psychopathology perspective. Dev. Psychopathol..

[23] Assed MM (2020). Facial emotion recognition in maltreated children: a systematic review. J. Child Fam. Stud..

[24] Cloitre M (2019). Emotion regulation mediates the relationship between ACES and physical and mental health. Psychol. Trauma. Theory Res. Pract. Policy.

[25] Gibb BE (2002). Childhood maltreatment and negative cognitive styles: a quantitative and qualitative review. Clin. Psychol. Rev..

[26] Schierholz A (2016). What mediates the link between childhood maltreatment and depression? The role of emotion dysregulation, attachment, and attributional style. Eur. J. Psychotraumatol..

[27] Chen M, Lacey RE (2018). Adverse childhood experiences and adult inflammation: findings from the 1958 British birth cohort. Brain Behav. Immun..

[28] Nurius PS, Logan-Greene P, Green S (2012). Adverse childhood experiences (ACE) within a social disadvantage framework: Distinguishing unique, cumulative, and moderated contributions to adult mental health. J. Prev. Intervent. Commun..

[29] Liu SR, Kia-Keating M, Nylund-Gibson K (2018). Patterns of adversity and pathways to health among White, Black, and Latinx youth. Child Abus. Negl..

[30] Chung EK (2016). Screening for social determinants of health among children and families living in poverty: a guide for clinicians. Curr. Probl. Pediatr. Adolesc. Health Care.

[31] Chandan JS (2020). Exploration of trends in the incidence and prevalence of childhood maltreatment and domestic abuse recording in UK primary care: a retrospective cohort study using ‘the health improvement network’database. BMJ Open.

[32] Schneider M, VanOrmer J, Zlomke K (2019). Adverse childhood experiences and family resilience among children with Autism spectrum disorder and attention-deficit/hyperactivity disorder. J. Dev. Behav. Pediatr..

[33] Baim, C. *The Drama Spiral: A Decision-Making Model for Safe, Ethical, and Flexible Practice when Incorporating Personal Stories in Applied Theatre and Performance.*10.1007/978-3-319-63242-1_4 (2017).

[34] Fancourt, D. et al. How do artistic creative activities regulate our emotions? Validation of the Emotion Regulation Strategies for Artistic Creative Activities scale (ERS-ACA). *PLoS One* **14**, e0211362 (2019). 10.1371/journal.pone.0211362 PMC6363280 30721269

[35] Zarobe L, Bungay H (2017). The role of arts activities in developing resilience and mental wellbeing in children and young people a rapid review of the literature. Perspect. Public Health.

[36] Robarts J (2006). Music therapy with sexually abused children. Clin. Child Psychol. Psychiatry.

[37] Pretorius G, Pfeifer N (2010). Group art therapy with sexually abused girls. South Afr. J. Psychol..

[38] Olff M (2015). Mobile mental health: a challenging research agenda. Eur. J. Psychotraumatol..

[39] Gulliver A, Griffiths KM, Christensen H (2010). Perceived barriers and facilitators to mental health help-seeking in young people: a systematic review. BMC Psychiatry.

[40] Lester S, Khatwa M, Sutcliffe K (2020). Service needs of young people affected by adverse childhood experiences (ACEs): a systematic review of UK qualitative evidence. Child. Youth Serv. Rev..

[41] Jackson DB, Testa A, Fox B (2021). Adverse childhood experiences and digital media use among U.S. children. Am. J. Preventive Med..

[42] Aboujaoude E (2020). Editorial: Digital interventions in mental health: current status and future directions. Front. Psychiatry.

[43] Liverpool S (2020). Engaging children and young people in digital mental health interventions: systematic review of modes of delivery, facilitators, and barriers. J. Med. Internet Res..

[44] Fitzgerald M, Ratcliffe G (2020). Serious games, gamification, and serious mental illness: a scoping review. Psychiatr. Serv..

[45] Anderson KM, Cook JR (2015). Challenges and opportunities of using digital storytelling as a trauma narrative intervention for traumatized children. Adv. Soc. Work.

[46] The Lancet. (2020). Child mental health services in England: a continuing crisis. Lancet.

